# Complete Genome Sequence of Solvent-Tolerant *Clostridium beijerinckii* Strain SA-1

**DOI:** 10.1128/genomeA.01310-14

**Published:** 2014-12-18

**Authors:** Jesse Noar, S. T. Makwana, José M. Bruno-Bárcena

**Affiliations:** Department of Plant and Microbial Biology, North Carolina State University, Raleigh, North Carolina, USA

## Abstract

We report the complete genome sequence of *Clostridium beijerinckii* SA-1, derived by directed evolution from *C. beijerinckii* NCIMB 8052, selecting for enhanced solvent tolerance. This sequence allows for accurate placement of SA-1 as *C. beijerinckii*, permits functional analyses of mutant phenotypes, and suggests methods for distinguishing SA-1 from its parent.

## GENOME ANNOUNCEMENT

*Clostridium beijerinckii* is a Gram-positive, obligately anaerobic solventogenic organism, of interest primarily for acetone-butanol-ethanol (ABE) fermentations. Taxonomic assignments of solventogenic species of *Clostridium* historically have experienced some turmoil, and strains originally classified as one species have often been reassigned to another (1).

In 1983, using stress-directed evolution with a series of increasing butanol concentrations, Lin and Blaschek isolated an especially butanol-resistant strain of *Clostridium*, originally designated an offspring of *C. acetobutylicum* ATCC 824 (2). This strain was named SA-1, deposited, and catalogued as ATCC 35702; later it was reclassified as a strain of *Clostridium beijerinckii*, an offspring of *C. beijerinckii* NCIMB 8052 (1). SA-1’s nutritional requirements were determined previously, and butanol resistance and delayed sporulation phenotypes have been observed (3). Other studies on SA-1 have evaluated its extracellular alpha-amylase and glucoamylase activity and the effects of butanol stress on its membrane fatty acid composition (4, 5). Reportedly, SA-1 is genetically malleable (6).

Sequencing the genome of *C. beijerinckii* SA-1 allows for definitive placement of this strain within the *C. beijerinckii* species. Identification of variations in SA-1’s sequence from that of its parent can guide functional physiological studies to ascertain the extent of SA-1’s butanol tolerance and solventogenic capacities, as well as other mutant phenotypes (3, 7); alternatively, such a project allows for the identification of errors in the original sequence of *C. beijerinckii* NCIMB 8052 (7). Finally, discovery of large variations between strains suggests a simple molecular method for distinguishing them from each other (7).

The genome sequence of *C. beijerinckii* SA-1 was determined using the Illumina Genome Analyzer IIx platform by the US Department of Energy Joint Genome Institute. Reads were assembled by comparison to the sequence of parent strain *C. beijerinckii* NCIMB 8052 (GenBank accession no. NC_009617), and algorithms MAQ (8) and BreakDancer (9) were used to find small and large variations from the reference sequence. PCR and Sanger dye-terminator sequencing (Eton Bioscience, Research Triangle Park, NC) were used to validate variations found *in silico*. Annotations were copied from the GenBank record of the genome of NCIMB 8052 and modified as necessary.

### Nucleotide sequence accession number.

The genome sequence of *C. beijerinckii* strain SA-1 (ATCC 35702) has been deposited in DDBJ/EMBL/GenBank under the accession no. CP006777. The version described in this paper is the first version.
